# Abstract of the Report of the Committee of the Academy of Medicine on Catheterization of the Air-Passages

**Published:** 1855-08

**Authors:** 


### SELECTIONS.

*Abstract of the Report of the Committee of the Academy of Medicine on Catheterization of the Air-Passages.*

This committee was appointed to report upon a paper read before the Academy of Medicine, by Dr. Horace Green, "On the Employment of Injections into the Bronchial Tubes, and into Tubercular Cavities of the Lungs." The committee consisted of Prof. Willard Parker, chairman, Drs. Alex. H. Stevens, Isaac Wood, J. Anderson, J. T. Metcalfe, B F. Barker, and John O. Stone. The following is an abstract of the majority report.

The committee met on five different occasions. At these different meetings thirty-eight patients, in the aggregate, were submitted by Dr. Green and others to the test of the operation of passing instruments into the air-passages, and three, of the injection of nitrate of silver into these passages.

I. *As to the practicability of the operation of passing instruments into the air-passages.*

This division of the report was considered under the following heads:—

1. *History of catheterism of the air-passages.*—The introduction of tubes into the air-passage is not a recent operation. It was recommended by Hippocrates, and seems to have been long practiced in preference to bronchotomy. Desault, among modern writers, also recommended it strongly. Mr. Ryland, in his treatise on Diseases of the Larynx, devotes a short section to this operation. Other authors allude to it as a matter of history.

2. *Results of experiments.*—The results of experiments confirm the records of history, as to the practicability of the operation of catheterizing the air-passages. In 11 cases it was performed to their entire satisfaction. The evidences on which they rely as proofs of its success are :—

1. *The detection of the tube in the cavity of the larynx by the finger passed into the mouth.*—This was done by several of the committee, and was demonstrative of the position of the tube in relation to the laryngeal openings.

2. *The symptoms manifested by the patient :*

The symptoms which distinguish the passage of an instrument into the trachea and œsophagus, may be thus contrasted :—

TRACHEA.

1. Suffusion of the face, rapidly increasing to turgescence and lividity.
2. Great anxiety and alarm ; not easily pacified.
3. Eyes wild, staring, overflowing with tears.
4. Cough violent and spasmodic.
5. Respiration greatly disturbed ; inspiration loud, hoarse, and croupy ; expiration attended with violent cough, ejection of bronchial mucus through the tube, and finally, full breathing through the instrument.
6. Voice extinguished, a hoarse whisper, interpreted with difficulty.
7. Retching slight.

ŒSOPHAGUS.

1. Suffusion of the face slight, rapidly subsiding, with cessation of retching and vomiting.
2. Little anxiety ; easily pacified.
3. Eyes natural ; slight suffusion from tears.
4. Little or no cough.
5. Respiration little, if at all disturbed ; occasional puffs of air through the tube.
6. Voice distinct ; often quite natural.
7. Retching and vomiting a common symptom.

So prominent and characteristic are the symptoms which the patient manifests on the successful introduction of the instrument into the air-passages, and so strikingly do they contrast with those witnessed when it enters the œsophagus, that the committee came to regard the rational signs as the surest criterion of the success of the operation. In but a single instance did they fail of being exhibited with characteristic intensity. In this case the patient had suffered from a syphilitic ulceration of the air-passages, and to this circumstance, together with his enfeebled condition, was attributed the absence of the severe symptoms. Dyspnœa and lividity of the face were the only evidence of the success of the operation. They therefore establish it as a rule, to which ordinarily there is no exception, that the rational signs above tabulated will differently distinguish the course which the instrument takes, whether into the laryngeal or œsophagus passages.

Other tests were employed, but none of them were found on trial free from objection.

The power of the patient to blow out a lighted candle, and to collapse and inflate at will, in respiration, a bladder attached to its free extremity, through the tube, has been much relied upon as a proof that the tube had entered the trachea. But it was found that when the tube was purposely passed into the œsophagus, this power was still retained, though in a much less degree.

The passage of two sponge-armed probangs into the throat, and by the withdrawal of the lower one, calculating the position of the other, whether in the œsophagus or trachea, did not, as far as experimented with, yield satisfactory results. For it was not proved to the satisfaction of the committee that the sponge-probang entered the larynx and trachea.

The sensations of the patient are reliable only when the tube has been repeatedly passed both into the trachea and œsophagus. In these cases patients have very promptly and correctly decided which passage the instrument was entering. This fact was strikingly illustrated in Mary Norton, on whom the operation was repeatedly performed before she was brought before the committee. On the first and second trials of the introduction of the tube, by Dr. Green, she shook her head, indicating that it had not entered the larynx. The operator, however, was satisfied on the last trial that the tube had penetrated the trachea, and that the sensations of the patient were not to be relied on, when she ejected a portion of the contents of the stomach through the tube, and thus demonstrated not only the position of the instrument, but equally the correctness of her sensation.

It appeared very evident, also, in the course of these experiments, that the opinion of the operator, as to the course which the instrument takes, is unreliable, when he trusts to his own senses, and disregards symptoms. They witnessed in two cases the fallacy of Dr. Green's opinion, as to the success of his experiment, though based upon so large an experience. In both instances, while positive that he had successfully passed the instrument into the trachea, the patient vomited through the tube, and thus demonstrated his error.

II. *Facility of the operation.*—While these experiments proved conclusively to the minds of the committee, the practicability of passing tubes into the air-passages, they also afforded opportunities of witnessing the facility with which the operation is performed under the different circumstances, and with different instruments. These were as follows:—

1. *Previous preparation of the patient.*—It is contended that the facility with which the instrument is introduced depends much upon the previous preparation of the patient, by frequent application of the nitrate of silver to the upper part of the larynx. To obviate this objection, the committee met twice at Dr. Green's office, to witness the experiment upon patients whom he was treating with local applications to these parts, and at the second visit at Bellevue Hospital, the patients were selected from a class which had been expressly prepared for the experiment, by the application of caustic to the throat from three to five times during the preceding week.

The results of these experiments, however, do not sustain the above assertion. The patients, on whom Dr. Green had been operating for six months, more or less, were as intolerant of the actual passage of the tube in the trachea, as those first submitted to trial. The case of Mary Norton was adduced. Although the tube in her case had been passed into the trachea half-a-dozen times previously to her coming before the committee, still she exhibited, in the most marked manner, all the evidences characteristic of the introduction of a foreign body into the air-passages, when the tube was successfully passed.

2. *Instruments Employed.*—The facility with which catheterism of the air-passages is performed seems to depend in a great degree upon the instruments used. Those employed in these experiments were: 1. A tube, consisting of Hutching's catheter, No. 10, with a wire stilet, and bent with a curvature corresponding to a circle of six inches in diameter. 2. A tube of the same size, slightly bent at its extremity. This was the instrument selected by Dr Green, and is the one which he is accustomed to use in practice. 3. A sponge armed probang, the sponge having a diameter of one half to three quarter of an inch. The result of the experiments with these several instruments was as follows:—

| | No. of trials. | Failed. | Succeeded. | *Per cent.* Failures. |
|---|---|---|---|---|
| Tube with large curve, | 19 | 8 | 11 | about 28 |
| Tube with small curve, | 37 | 24 | 3 | " 92 |
| Sponge probang, | 18 | 18 | 0 | 100 |

From these experiments it appears that the instrument best adapted to succeed in catheterism of the air-passages, it is a tube having a large curve, or one shaped like a common catheter; while that least adapted to enter the trachea is the sponge-armed probang.

1. *The Tube with a Large Curve.*—This instrument was introduced into the trachea eleven times, to the entire satisfaction of the Committee. The fact that the operators were successful eleven times in nineteen trials, proves that this tube can be introduced with considerable certainty into the air-passage.

2. *The Tube with a Small Curve.*—This is the instrument selected by Dr. Green for catheterization of the air-passages, and is that which he uses in practice. In these experiments he was the only one who employed it, and the result shows that even in the hands of the most skillful practitioner, it failed of entering the air-passage in about 92 per cent. of trials.

3. *The Sponge-Probang.*—This instrument afforded the least satisfactory results of any used. Notwithstanding the most persevering efforts with the whalebone slightly bent, as used by Dr. Green, and with patients who quietly submitted to the test of experiment, the results were entirely negative. In no instance did it satisfactorily enter the trachea. In two instances, with the whalebone curved like a common catheter, the sponge was thought to have entered the larynx; but with repeated efforts it could not be passed between and beyond the vocal chords. The suffocation was so great each time as to compel a withdrawal of the instrument.

III. *The Utility of Injections into Tubercular Cavities of Lungs.*—This portion of the report was brief. As the Committee had no evidence that injection could be thrown into tubercular cavities of the lungs, they refused to discuss, on merely theoretical grounds, the question of their utility. One of the three cases injected with nitrate of silver died within twenty-four hours after the operation, not having recovered from its immediate effects. A letter was read from the physician who made the autopsy, describing the condition of the air-passages. The mucous membrane had the appearance of a recent application of the caustic.

*Conclusions.*—1. Catheterism of the air-passages dates its history from the time of Hippocrates.

2. The best evidence of the successful passage of an instrument into the air-passages, are rational signs.

3. The facility of the operation depends upon the kind of instrument used—the tube having a large curve being best, and the sponge-probang least adapted to enter the trachea.

4. That there is no reliable evidence, in the opinion of the Committee, that the sponge-probang has been passed through and beyond the vocal chords.

5. That there is no sure evidence that an instrument can be passed *at will* into the right or left bronchial divisions.

6. That the great majority of instances where injections are supposed to have been thrown into the lungs through a tube, they have passed directly into the stomach.

7. That as regards the utility of injections of nitrate of silver into the lungs, the facts thus far developed in the experiment of the Committee, lead them to regard the operation as one fraught with danger as well as difficult.

This report was signed by Prof. Parker, Chairman, Drs. Wood, Metcalf, and Stone; Dr. Anderson subscribed to all but that bearing upon the utility of injections: this he did not think sufficiently investigated; Dr. Stevens, who witnessed but a few experiments, made a short report. Prof. Barker made a lengthy minority report.—*N. Y. Med. & Col. Sciences.*

